# Comparison of Five Oral Cannabidiol Preparations in Adult Humans: Pharmacokinetics, Body Composition, and Heart Rate Variability

**DOI:** 10.3390/ph14010035

**Published:** 2021-01-06

**Authors:** Natasha N. Bondareva Williams, Taylor Russell Ewell, Kieran Shay Struebin Abbotts, Kole Jerel Harms, Keith A. Woelfel, Gregory P. Dooley, Tiffany L. Weir, Christopher Bell

**Affiliations:** 1 Department of Health and Exercise Science, Colorado State University, Fort Collins, CO 80523-1582, USA; Natasha.Williams@colostate.edu (N.N.B.W.); Taylor.Ewell@colostate.edu (T.R.E.); Kieran.Abbotts@colostate.edu (K.S.S.A.); kharms@rams.colostate.edu (K.J.H.); 2 Caliper Foods, Commerce City, CO 80022-3970, USA; keith.woelfel@caliper.life; 3 Department of Environmental and Radiological Health Sciences, Colorado State University, Fort Collins, CO 80523-1680, USA; Gregory.Dooley@colostate.edu; 4 Department of Food Science and Human Nutrition, Colorado State University, Fort Collins, CO 80523-1571, USA; Tiffany.Weir@colostate.edu

**Keywords:** bioavailability, fat free mass, autonomic

## Abstract

Data supporting the physiological effects of cannabidiol (CBD) ingestion in humans are conflicting. Differences between CBD preparations and bioavailability may contribute to these discrepancies. Further, an influence of body composition on CBD bioavailability is feasible, but currently undocumented. The aims of this study were to: (1) compare the pharmacokinetics of five oral CBD preparations over 4 h; (2) examine the relationship between body composition and CBD pharmacokinetics; and, (3) explore the influence of CBD on heart rate variability. In total, five preparations of CBD, standardized to 30 mg, were orally administered to 15 healthy men and women (21–62 years) in a randomized, crossover design. Prior to and 60 min following CBD ingestion, heart rate variability was determined. Body composition was assessed using dual energy X-ray absorptiometry. Peak circulating CBD concentration, time to peak concentration, and area under the curve was superior in a preparation comprising 5% CBD concentration liquid. Fat free mass was a significant predictor (*R*^2^ = 0.365, *p* = 0.017) of time to peak concentration for this preparation. Several heart rate variability parameters, including peak frequency of the high frequency band, were favorably, but modestly modified following CBD ingestion. These data confirm an influence of CBD preparation and body composition on CBD bioavailability, and suggest that acute CBD ingestion may have a modest influence on autonomic regulation of heart rate.

## 1. Introduction

Cannabidiol (CBD), a non-psychoactive component of *Cannabis sativa* L., has been purported to have a variety of beneficial physiological effects including but not limited to, pain relief, anti-anxiety, anti-seizure, anti-depressant, anti-oxidant, and anti-inflammatory [1]. Despite the enthusiasm of the CBD industry for these claims, the empirical evidence supporting favorable physiological responses is inconsistent [2,3]. These discrepancies may in part be explained by differences in CBD bioavailability when administered orally [4], that in turn may be influenced by non-standardized CBD formulation, and/or the body size and composition of the recipient. With regards to the former, differences in formulation might include water vs. lipid solubility, administration as a food vs. a beverage, co-administration with additional ingredients, and/or preparation as a ready-made product vs. a powder to be mixed with liquid by the consumer before ingestion. Once ingested, the resultant circulating CBD concentration will be influenced by rates of absorption from the gut, breakdown during first pass metabolism, and potentially by the body size and composition of the consumer. For example, lean mass is positively associated with total blood volume [5,6]. Thus, it is feasible that adults with a higher lean mass may demonstrate lower circulating CBD concentrations on account of a larger blood volume in which to dilute the CBD. Alternatively, fat mass may influence CBD absorption as CBD is lipid soluble and can therefore potentially accumulate in adipose tissue in a manner similar to that previously reported for Δ^9^-tetrahydrocannabinol, a psychoactive component of *Cannabis sativa* L. [7,8]. Although intuitive, the influence of body size and composition on CBD pharmacokinetics is currently undocumented. The purpose of the current study was to compare the pharmacokinetics of five oral CBD preparations over 4 h, and to examine the relationship between body composition and CBD pharmacokinetics.

One of the inconsistent physiological responses to CBD administration is the cardiovascular response [9]. Some studies have reported changes in heart rate and/or blood pressure following acute CBD consumption [10,11] while others have reported no measurable effect [12,13]. Changes in heart rate (in any direction) are almost always mediated by changes in the sympathovagal balance; this balance is typically described via heart rate variability. Heart rate variability refers to the inconsistency within the periods of time separating consecutive cardiac cycles. It is considered to have clinical relevance as it is able to predict future cardiac events and mortality [14,15,16]. To our knowledge, only one other study has examined the influence of CBD (specifically, a hemp-oil extract containing CBD) on heart rate variability in adult humans [17], and this study reported on short-term CBD use (3 and 6 weeks) and not an acute response. Accordingly, an additional purpose of the current study was to explore the acute influence of CBD ingestion on heart rate variability.

## 2. Results

### 2.1. Participants

**Figure 1:**
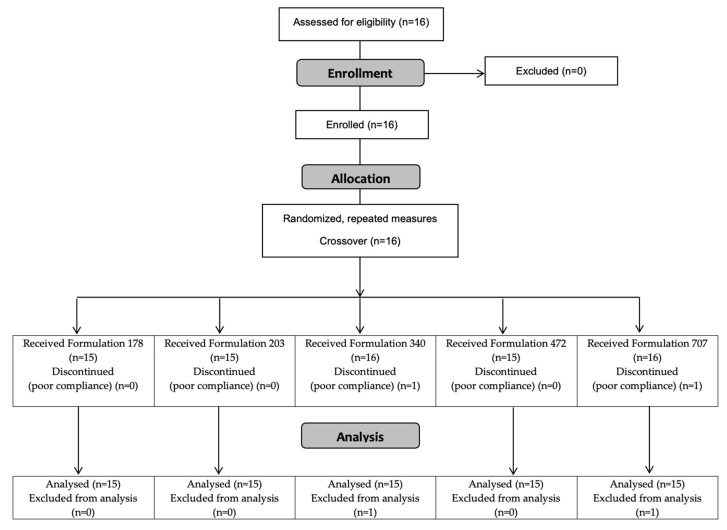
Consolidated Standards of Reporting Trials (CONSORT) flow diagram.

The progress of all participants throughout the trial (from screening and enrollment through to completion) is presented in Figure 1. A total of 16 participants were enrolled in the study. Due to repeated scheduling difficulties, one participant was removed from the study and replaced with a new recruit; a total of 15 participants completed all procedures in a crossover design. Selected physiological characteristics are presented in Table 1. Consistent with inclusion and exclusion criteria, the physiological characteristics were unremarkable, and typical of a broad sample of adults, free from overt cardio-metabolic disease.

### 2.2. Circulating CBD and Pharmacokinetics

**Figure 2:**
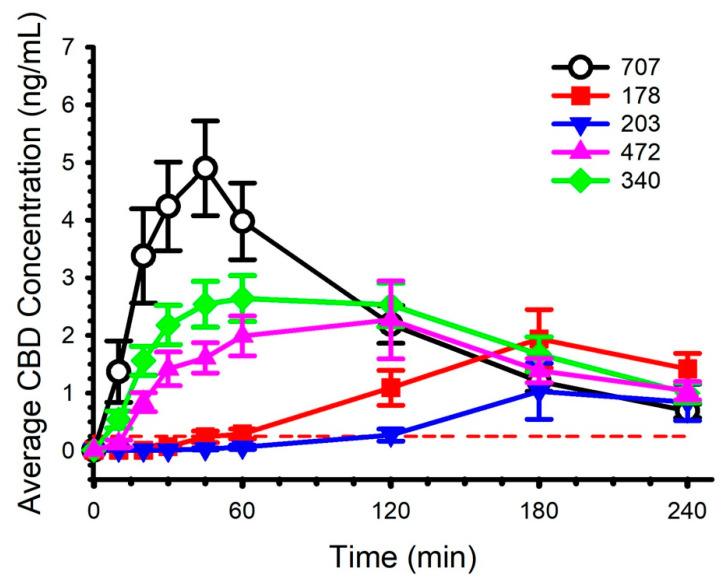
Circulating cannabidiol (CBD) concentration following ingestion of five different preparations. Dose was standardized to 30 mg. Limit of quantitation was 0.25 ng/mL and is represented by the red dashed line. Data are mean and standard error.

Each of the CBD preparations contained a standardized CBD dose of 30 mg. The characteristics of each of the preparations are described in Table 2. Circulating concentrations of CBD and the calculated pharmacokinetics are presented in Figure 2 and Table 3, respectively. Baseline (Time 0) concentrations for all CBD preparations, for all participants, were below the limit of quantitation. By the final blood collection (4 h), average concentration was above the limit of quantitation for each preparation, but not for each participant (1 participant below for 178, 2 participants below for 203, 0 participants below for 340, 1 participant below for 472, and 2 participants below for 707). Informal visual inspection of Figure 2 suggested preparation 707 evoked the highest circulating CBD concentration, the shortest time to maximal concentration, and the greatest area under the curve during the blood collection period. Formal statistical analysis, detailed in Table 3, supported several of these interpretations, although not all comparisons between preparation 707 and the other preparations attained statistical significance (*p* < 0.05). It was not possible to calculate some pharmacokinetic parameters (i.e., AUC_inf_, t_½_, K_e_ and V_d_) for all of the CBD preparations on account of insufficient values above the limit of quantitation during the initial 1–2 h of blood collection.

### 2.3. Body Composition

Within each CBD preparation, there was considerable variability in the calculated pharmacokinetic parameters. Correlation analysis revealed several associations between some of these parameters and selected physiological characteristics. For example, the T_max_ for 707 was correlated with height (r = −0.44, *p* = 0.099), bone mineral content (r = −0.53, *p* = 0.04), lean mass (r = −0.60, *p* = 0.017), fat free mass (r = −0.60, *p* = 0.017), body mass (r = −0.52, *p* = 0.048), and %body fat (r = 0.46, *p* = 0.082). When these variables were considered together in forward stepwise regression, only fat free mass remained a significant predictor (R^2^ = 0.365, *p* = 0.017) of T_max_. Similarly, T_max_ for 178 was correlated with body mass index (r = 0.63, *p* = 0.016), bone mineral content (r = 0.61, *p* = 0.021), fat free mass (r = 0.46, *p* = 0.095), and body mass (r = 0.47, *p* = 0.091). Forward stepwise regression revealed that the only significant predictor was body mass index (R^2^ = 0.397, *p* = 0.016). The only other relations of significance were height with 178 AUC_0-4_ (r = 0.58, *p* = 0.03), and age with 707 C_max_ (r = 0.64, *p* = 0.011) and 707 AUC_0-4_ (r = 0.58, *p* = 0.024).

### 2.4. Heart Rate Variability

Heart rate variability was assessed immediately prior to and 60 min following CBD ingestion. Circulating CBD concentrations for preparations 178 and 203 were below the limit of quantitation at 60 min, thus heart rate variability data associated with these preparations were excluded from statistical analysis. The heart rate variability data are presented in Table 4. There were main effects of time (all *p* < 0.05) for heart rate (decreased), R-to-R interval (increased), peak frequency of the high frequency band (decreased), Poincaré plot standard deviation perpendicular the line of identity (increased), and Poincaré plot standard deviation along the line of identity (decreased). In addition, there were several parameters with main effects of time that did not attain statistical significance (0.05 < *p* < 0.08) including, baseline width of the R-to-R interval histogram (increased), and the ratio of Poincaré plots standard deviation perpendicular to along the line of identity (decreased). There were no time x CBD preparation interactions (all *p* > 0.10). There were no appreciable changes in heart rate and blood pressure across the 4 h of data collection (Table 5).

## 3. Discussion

The goals of the current study were to compare the pharmacokinetics of five oral CBD preparations over 4 h, to examine the relationship between body composition and CBD pharmacokinetics, and to explore the influence of acute CBD ingestion on heart rate variability. Our primary findings were: (1) compared with most of the other preparations, the preparation comprising 5% CBD concentration liquid (preparation 707; Table 2) evoked the fastest T_max_, and the greatest C_max_; (2) within each CBD preparation, there was considerable variability in the calculated pharmacokinetic parameters; some of the variability for some of the preparations could be explained by body size and composition; and, (3) CBD had only a modest effect on some of the parameters used to describe heart rate variability.

The high degree of variability in CBD absorption and the resulting pharmacokinetic parameters following CBD ingestion have been previously described [4,12,18,19,20]. Potential explanations for variable, and often poor, CBD absorption have pertained to properties of the CBD itself (e.g., poor water solubility) and also variability brought about by the significant first pass metabolism it must endure following oral ingestion. To counter these potential absorption hurdles, CBD preparations have been modified, with mixed success, in a variety of ways, including generation of synthetic CBD [20], creation of water-soluble CBD powders [12], development of self-emulsifying delivery systems [18,20], and encapsulation of CBD within gelatin matrix pellets [21] and liposomes [22]. One important consideration when evaluating CBD formulations is the pharmacokinetic goal and intended use of the CBD. For example, if the indication for the CBD is to treat acute pain, then a faster T_max_ and higher C_max_ may be desirable, and also may help to decrease the risk of overdose due to premature repeat self-administration. Alternatively, as a chronic treatment for anxiety, a larger AUC may be preferrable if a user follows a regular dosing schedule. The CBD preparations under investigation in the current study generated a wide range of values for pharmacokinetic parameters (Table 3). Preparation 707 appeared to be the superior preparation as, compared with most of the others, it generally evoked the fastest T_max_, greatest C_max_, and largest AUC. Perhaps more important than comparisons within the current study are comparisons of the preparations to others described within the literature. In this regard, a very thorough review of CBD pharmacokinetics was published in 2018 [19]. We have supplemented this published information to include data from the current study, and other data published since the release of the original review (Table 6). An important caveat to these comparisons is that the dose of CBD administered across the reviewed studies ranged between 5 and 6000 mg; the influence of CBD dose on pharmacokinetics, especially AUC, has been previously described [19,23], thus direct comparisons to appreciably smaller/larger doses are problematic. Further, these are informal and not definitive statistical/quantifiable comparisons. With these caveats in mind, preparation 707 from the current study appears to have the fastest T_max_, but a smaller C_max_ when compared with the Self-Emulsifying Drug Delivery System, despite the latter comprising a slightly smaller dose (25 mg) [18]. The favorable CBD bioavailability of the Self-Emulsifying Drug Delivery System was attributed to augmented solubility within the gastrointestinal tract [18]. A similar rationale was employed during the development of preparation 707; some of the components of the preparation, such as gum arabic and medium-chain triglycerides (MCTs) have been shown to improve bioavailability of dietary supplements [24,25] and may have contributed to the superior performance in the current study, and its favorable comparison to the other published formulations.

Another important consideration for CBD use is terminal elimination half-life. In the context of pharmacokinetic research, undetectable baseline circulating CBD concentrations can be critical for valid derivation of pharmacokinetic parameters. The terminal elimination half-life of CBD administered to fasted humans varies greatly (between 6 and 32 h) [37,38] depending on mode of administration and CBD preparation. This variability is further illustrated by the K_e_ values (i.e., the rate of CBD removal from the body) presented in Table 3 and Table 6. In the current study, baseline (Time 0) concentrations for all CBD preparations, for all participants, were below the limit of quantitation. This provides support for our rationale for a minimum 72 h “washout” period between consecutive laboratory visits, although it should be noted that other studies have employed considerably longer duration (14 days) CBD abstention periods between repeated study visits [18].

An influence of body size and composition on CBD pharmacokinetics has been previously speculated [18] but to our knowledge is currently undocumented. In the current study we used DEXA to quantify body composition. Some of the inter-personal variability, for some of the preparations, could be explained by body size and composition as reflected by correlations between several of the DEXA-derived variables and CBD pharmacokinetic parameters. It is not uncommon for DEXA-derived variables to be interrelated (for example, high fat free mass is often associated with high body mass), thus we employed stepwise regression to further explore potential relations between body composition and CBD pharmacokinetics. With respect to our superior CBD preparation, preparation 707, fat free mass was a significant predictor of T_max_ (r = −0.60, *p* = 0.017).

Somewhat counter-intuitively, the association was negative, implying that a larger fat free mass was associated with a faster T_max_. In light of the positive association between fat free mass and total blood volume [5,6], it may seem unlikely that peak concentration is reached earlier in adults with greater blood volumes. Circulating concentrations of all substances, including endogenous hormones and active ingredients in dietary supplements, are determined by a combination of rate of entry and rate of clearance from the blood. The shorter time to peak concentration in adults with greater fat free mass may reflect a greater rate of clearance by metabolically active and relatively well-perfused tissues (such as skeletal muscle). Noteworthy, fat free mass did not predict C_max_ or AUC for preparation 707. Alternatively, body mass index was positively associated with T_max_ for preparation 178 (r = 0.63, *p* = 0.016). The preparation 178 T_max_ was among the slowest of the compared preparations (approximately 3.5 hours); it is feasible that for preparations absorbed more slowly (i.e., slower rate of entry into the blood), body size (and presumably blood volume) may contribute to time to peak circulating concentration. From a broader perspective, CBD bioavailability may be determined not only by preparation/formulation, but perhaps also by body size and composition. The implication is that future dosing guidelines may better serve users of CBD when additional consideration is given to expected rates of CBD absorption based on preparation/formulation characteristics (e.g., presence/absence of ingredients used to promote solubility), requirements of intended use (e.g., acute needs from a single dose vs. chronic repeated dosing), and the body size and composition of the user. Dosing guidelines should be a focus of future research as we are unable to make definitive recommendations based on our current data.

An additional goal of the current study was to explore the influence of acute CBD ingestion on heart rate variability, the inconsistencies in the periods of time separating consecutive cardiac cycles. It is an important predictor of future cardiac events [14,15] and, in some instances, longevity [16]. While there are many methods and different parameters used to quantify heart rate variability, generally speaking, greater variability is considered desirable. To our knowledge, this is the first exploration of a potential influence of acute CBD on heart rate variability in adult humans. Only one previous study has examined the short-term influence of CBD (administered as a hemp oil extract) on heart rate variability [17] but the experimental design did not include an acute measurement. Other studies have compared heart rate variability in users and non-users of *Cannabis sativa* L. (i.e., CBD plus Δ^9^-Tetrahydrocannabinol and other potentially active ingredients), thus making assertions as to the independent influence of CBD difficult [39]. In the current study, acute CBD ingestion decreased heart rate, peak frequency of the high frequency band, and Poincaré plot standard deviation along the line of identity, and increased R-to-R interval and Poincaré plot standard deviation perpendicular to the line of identity (Table 4). Poincaré plots are a useful tool in the heart rate variability analysis arsenal; they are often able to recognize patterns and rhythms in the R-to-R interval data that are sometimes overlooked using spectral analysis [40,41]. From an interpretation perspective, the standard deviation perpendicular to the line of identity reflects short-term heart rate variability and is thought to be regulated by parasympathetic (vagal) input, while the standard deviation along the line of identity reflects total heart rate variability and is determined by a combination of sympathetic and parasympathetic input [40]. When considered together, the ratio of these two variables (i.e., the SD2/SD1 ratio; Table 4) represents sympathovagal balance. In light of the opposing direction of change in these Poincaré plot data, in addition to the small magnitude of change in the other heart rate variability parameters, it appears likely that acute CBD evokes only a modest, and physiologically irrelevant effect on heart rate variability, at least when studied at rest. In the short-term study [17], neither 3 nor 6 weeks of daily CBD ingestion influenced heart rate variability measured at rest or during mental stress activities. One important point worthy of emphasis in the current study—our primary focus was the pharmacokinetics of the different CBD preparations and the potential interaction with body size and composition. Accordingly, we did not include a placebo-control within our experimental design. Thus, any conclusions with respect to our exploration of the acute influence of CBD on heart rate variability must be considered with caution. For example, it is plausible that CBD does not exert any acute influence on heart rate variability and we are simply reporting on the influence of 60 min of semi-recumbent rest.

CBD may or may not have appreciable, and potentially favorable effects on physiological function. Conflicting empirical evidence describing the response to CBD can be partially explained by considerable inconsistencies in its bioavailability. In the current study we identified a CBD preparation that compared well with other preparations described in the literature [19], and with the other preparations incorporated within our design. Further, we demonstrate that some of the pharmacokinetic parameters of this superior preparation were influenced by body size and composition. These findings highlight the need to optimize CBD preparations and personalized dosing strategies in order to confirm/refute the physiological relevance of CBD as a potential therapeutic. Finally, we document, for the first time, a modest effect of acute CBD on selected parameters of resting heart rate variability in healthy adults, alleviating any potential concerns that CBD may unfavorably impact autonomic regulation of heart rate.

## 4. Materials and Methods

A randomized, double-blind, repeated measures cross-over study design was employed. The institutional review board at Colorado State University reviewed and approved all procedures in accordance with the principles established in the Declaration of Helsinki. Written informed consent was provided by all participants prior to commencement of any study activity.

### 4.1. Participants

Healthy adult men and women were invited to participate. Inclusion criteria included aged 18 years and over, body mass greater than 50 kg, absence of any known gastrointestinal or metabolic disease, and willing to refrain from all products containing CBD for 3 days prior to each study visit. Exclusion criteria included pregnancy, breastfeeding, known food allergies, autoimmune disorders, celiac disease, inflammatory bowel disease, gastrointestinal cancers, and history of diabetes. In addition, anyone who reported experiencing a previous adverse reaction to ingesting products containing *Cannabis sativa* L. were excluded from participation.

### 4.2. Protocol Overview

Following a screening visit consisting of a review of medical history and assessment of body composition, participants reported to the laboratory on five separate mornings in a crossover design. In light of the considerable variability in CBD absorption and the resulting pharmacokinetic parameters following CBD ingestion [4,12,18,19,20], a crossover design was employed in an attempt to minimize the influence of inter-individual variability. Each morning began with a baseline blood sample and determination of blood pressure, and heart rate variability. Participants then ingested one of five CBD preparations (described below). Venous blood was sampled over the next 4 hours. Heart rate variability was reassessed after one hour. Heart rate and blood pressure were also recorded over the next four hours.

### 4.3. Procedures

Participant screening comprised completion of a detailed medical history questionnaire and assessment of body size and composition using a physician’s digital scale and dual energy X-ray absorptiometry (Hologic, Discovery W, QDR Series, Bedford, MA, USA) as previously described [42].

The remaining five laboratory visits were identical in all aspects except for the CBD preparation that was administered. Participants reported to the laboratory on five separate mornings, each preceded by a 12 h fast. The terminal elimination half-life of CBD administered to fasted humans varies between 6 and 32 h [37,38]; to facilitate negligible baseline circulating CBD concentrations in each participant at the start of each study visit, every visit was preceded by a minimum 72 h abstention from all products containing CBD. The time of arrival was kept constant for each participant. On arrival, participants were instrumented for measurement of heart rate (3-lead electrocardiogram (ECG)) and blood pressure (auscultation) using a physiological monitor (IntelliVue MP5 Patient Monitor, Philips Healthcare, Andover, MA, USA); a venous catheter was introduced to an antecubital vein.

Heart rate variability was determined immediately prior to, and 60 min following CBD ingestion. During 11 min of paced-breathing (metronome: 6 breaths per minute) raw ECG signals were recorded using a personal computer and analogue-to-digital convertor (WinDaq, Dataq Instruments Inc., Akron, OH, USA) [43] and analyzed offline using commercially available software (Kubios HRV), as previously described [44,45]. The first minute was considered habituation and was discarded from the analysis. Additional measurements of heart rate and blood pressure were made prior to, and 30, 60, 120, 180 and 240 min after CBD ingestion. All measurements and recordings were completed in a temperature-controlled (20–22 °C), dimly lit room. During the measurements and recordings, participants were situated on a medical bed, in a comfortable, semi-recumbent posture.

Venous blood (~9 mL) was collected prior to, and 10, 20, 30, 45, 60, 120, 180, and 240 min after CBD ingestion. Blood was immediately transferred to chilled tubes containing ethylenediaminetetraacetic acid (EDTA). Plasma aliquots (1 mL) were separated from each of the samples and stored at −70 °C for later analysis.

Then, 90 min following CBD ingestion, participants were provided with a standardized breakfast consisting of bagel sandwich and choice of a non-alcoholic beverage. The breakfast was different between participants but was constant within laboratory visits (i.e., whatever a participant ate during the first visit, they ate for all of the subsequent visits).

### 4.4. Cannabidiol Preparations

In total, five different CBD preparations were provided by Caliper Foods (Commerce City, CO, USA). Each of the preparations contained a standardized CBD dose of 30 mg. The characteristics of each of the preparations are described in Table 2. Broadly speaking, the CBD preparations differed in their solubility (i.e., water vs. lipid), the concentration of the CBD powder, and additional ingredients used in each specific formulation. All preparations were administered in 227 mL (8 oz) of water and were consumed within ~30 s of administration.

### 4.5. Cannabidiol Analysis

Reagents and supplies: CBD and CBD-D3 were purchased from Cerilliant (Round Rock, TX, USA). Water and acetonitrile (LC-MS grade) were obtained from Millipore (Burlington, MA, USA) and formic acid (LC-MS-grade) from Sigma-Aldrich (St. Louis, MO, USA). Bond Elut dSPE Universal Sorbent was purchased from Agilent Technologies (Santa Clara, CA, USA). Chromatography was performed with a Kinetex Phenyl Hexyl column (3.0 mm × 50 mm, 2.6 μm) purchased from Phenomenex Inc. (Torrance, CA, USA).

Plasma samples were prepared for LC-MS/MS analysis by protein precipitation and dispersive solid phase extraction. In total, 200 μL of sample was mixed with 400 μL of ice-cold acetonitrile containing 20 ng/mL CBD-D3 in a microcentrifuge tube and vortexed for 30 s to precipitate proteins. Next, 200 mg of dSPE sorbent was added, vortexed for 30 s, and centrifuged for at 14,000 rpm for 5 min. Sample supernatants were then transferred to an autosampler vial for LC-MS/MS analysis.

Samples were analyzed with an Agilent 1290 UHPLC coupled to an Agilent 6460 triple quadruple mass spectrometer equipped with an Agilent Jet Stream electrospray ionization source (Agilent, Santa Clara, CA, USA). Cannabinoids were first chromagraphically separated on a Phenomenex Phenyl Hexyl column (3.0 mm × 50 mm, 2.6 μm) held at 40 °C. A sample volume of 10 μL was injected and a mixture of water with 0.1% formic acid (A) and acetonitrile with 0.1% formic acid (B) at a flow rate of 0.4 mL/min. The gradient elution used was 40% B for 0.5 min, increasing to 100% B at 2 min, and held at 100% B for 1.5 min. The ionization source conditions used were as follows: positive polarity, nebulizer 45 psi; gas flow of 10 L/min at 300 °C; sheath gas flow of 12 L/min at 375 °C. The ion transitions monitored for CBD were 315.2 → 193.1/123 m/z and 318.2 → 196.1/123.1 m/z for CBD-D3. CBD was confirmed by retention time and the product ion ratio correlation between the sample peaks and corresponding standards (±20%). The limit of detection and limit of quantitation for CBD in this analysis was 0.1 ng/mL and 0.25 ng/mL, respectively. The data collection and processing were performed by using Agilent MassHunter Quantitative software (v.B.08.01). Quantitative analysis was performed with linear regression using a 6-point calibration curves from 0.25 ng/mL to 50 ng/mL.

### 4.6. Pharmacokinetic Analysis

Pharmacokinetic analysis of the circulating concentrations of CBD for each of the preparations was completed using dedicated software (Phoenix WinNonlin v8.2, Certara, NJ, USA). Values below the limit of quantitation were classified as “missing”. Areas under the CBD concentration curves were calculated using the trapezoidal method.

### 4.7. Statistical Analysis

All data, unless otherwise stated, are expressed as mean and standard deviation. Statistical calculations were performed using dedicated software (Prism v8.4.3, GraphPad Software, San Diego, CA, USA). Differences in the pharmacokinetic properties between the CBD preparations were examined using 1-way analysis of variance mixed-effect models with Tukey tests employed to further explore identified main effects. Similarly, differences in the characteristics describing heart rate variability before/after CBD ingestion were also examined using 1-way analysis of variance mixed-effect models, and Tukey test when appropriate. Relations between CBD pharmacokinetic parameters and body size and composition values were explored using Pearson correlations, and further examined with forward stepwise regression when pharmacokinetics parameters were correlated with multiple body composition variables (SigmaStat 3.0, Systat Software Inc., San Jose, CA, USA). The level of statistical significance was set at *p* < 0.05.

## Figures and Tables

**Table 1:** Selected physiological characteristics of study participants.

Characteristic	Mean ± SD	Range
Sex (M/F)	9/6	-
Age (years)	29 ± 11	21–62
Height (cm)	174 ± 9	155–191
Body Mass (kg)	75.5 ± 13.8	58.6–100.1
Body Mass Index (kg/m^2^)	24.6 ± 3.5	18.8–31.4
Fat Mass (kg)	19.4 ± 4.8	12.0–30.1
Body Fat (%)	26.2 ± 6.6	18.0–40.7
Lean Mass (kg)	53.7 ± 12.3	33.3–70.8
Bone Mineral Content (kg)	2.4 ± 0.4	1.6–2.9

**Table 2:** Features of the cannabidiol (CBD) preparations.

Code	Preparation	Composition and Administration
178	CBD Tincture Base	MCT Oil droplet containing CBD isolate; administered in 227 mL (8 oz) of water
203	CBD Powder in Water	CBD as powder, suspended in reverse osmosis water; administered in 227 mL (8 oz) of water
340	20% CBD Concentration Liquid	Reverse osmosis water, CBD, MCT oil, quillaja extract; administered in 227 mL (8 oz) of water
472	5% CBD Concentration Powder	Water soluble CBD, sorbitol, modified food starch, natural flavors, MCT; oil administered in 227 mL (8 oz) of water
707	5% CBD Concentration Liquid	Reverse osmosis water, gum arabic, CBD, MCT oil, citric acid; administered in 227 mL (8 oz) of water

All preparations standardized to 30 mg of CBD. MCT: medium-chain triglyceride.

**Table 3:** Pharmacokinetic parameters.

Parameter	178	203	340	472	707
T_max_ (h)	3.29 ± 0.61 ^a,b^ *n* = 14	3.39 ± 0.65 ^c^ *n* = 13	1.28 ± 0.62 ^a^ *n* = 15	1.53 ± 1.02 ^b^ *n* = 15	0.70 ± 0.23 ^a,c^ *n* = 15
C_max_ (ng/mL)	2.20 ± 1.88 ^a^ *n* = 14	1.29 ± 1.93 ^b,c^ *n* = 13	3.54 ± 1.65 ^b^ *n* = 15	2.88 ± 2.48 ^d^ *n* = 15	5.57 ± 3.32 ^a,c,d^ *n* = 15
AUC_0-4_ (h × ng/mL)	4.58 ± 3.88 ^a^ *n* = 14	2.30 ± 2.77 ^b,c^ *n* = 13	7.81 ± 3.91 ^a,b^ *n* = 15	6.32 ± 4.57 *n* = 15	9.12 ± 5.21 ^c^ *n* = 15
AUC_0-inf_ (h × ng/mL)	- *n* = 0	- *n* = 0	13.81 ± 8.2*n* = 9	9.96 ± 8.11 *n* = 8	10.77 ± 5.71 *n* = 15
t_½_ (h)	- *n* = 0	- *n* = 0	2.20 ± 1.14 *n* = 9	5.18 ± 7.07 *n* = 8	1.42 ± 0.52 *n* = 15
K_a_ (1/h)	0.32 ± 0.24 *n* = 5	0.24 ± 0.00 *n* = 2	1.19 ± 0.80 *n* = 15	1.87 ± 2.23 *n* = 14	1.43 ± 0.65 *n* = 12
K_e_ (1/h)	- *n* = 0	- *n* = 15	0.40 ± 0.21 *n* = 9	0.27 ± 0.16 ^a^ *n* = 8	0.56 ± 0.22 ^a^ *n* = 15
V_d_ (L)	- *n* = 0	- *n* = 0	7428 ± 2232 *n* = 9	20,178 ± 9989 ^a^ *n* = 8	8024 ± 6630 ^a^ *n* = 15

Data are mean and SD. Limit of quantitation: 0.25 ng/mL. Values below limit of quantitation were classed as “missing”. *n*: number of observations used to calculate parameter. T_max_: the time to maximum concentration. C_max_: the maximum concentration. AUC_0-4_: the area under the curve representing total cannabidiol exposure between 0 and 4 h. AUC_0-inf_: an estimate of the total exposure to cannabidiol over time. t_½_: the amount of time it takes to decrease the circulating concentration to half of its initial value. K_a_: the rate at which the cannabidiol is absorbed into the body. K_e_: the rate at which the cannabidiol is removed from the body. V_d_: the volume of distribution, an estimate of the degree to which cannabidiol is distributed in the body tissue vs. the plasma. Values sharing the same superscript letter are different (*p* < 0.05).

**Table 4:** Heart rate variability prior to and 60 min following 30 mg cannabidiol ingestion.

Parameter	178		203		340		472		707	
	0	60	0	60	0	60	0	60	0	60
Time Domain										
HR (b/min) ^a^	57 ± 9	56 ± 10	61 ± 10	59 ± 10	58 ± 11	55 ± 10	58 ± 10	56 ± 9	57 ± 9	55 ± 9
R-to-R_Int_ (ms) ^a^	1076 ± 173	1110 ± 194	1011 ± 183	1049 ± 200	1061 ± 190	1113 ± 187	1069 ± 190	1106 ± 195	1075 ± 170	1119 ± 180
SDNN (ms)	112 ± 50	115 ± 57	126 ± 53	129 ± 53	126 ± 53	125 ± 46	133 ± 64	132 ± 53	123 ± 55	129 ± 47
RMSSD (ms)	100 ± 43	108 ± 56	118 ± 57	116 ± 50	113 ± 47	117 ± 41	121 ± 60	125 ± 55	113 ± 54	126 ± 49
R-to-R Triangular Index (ms)	19 ± 8	19 ± 6	17 ± 5	21 ± 6	21 ± 7	22 ± 8	22 ± 8	22 ± 10	21 ± 7	20 ± 8
TINN (ms) ^b^	466 ± 174	525 ± 237	537 ± 167	605 ± 188	592 ± 252	624 ± 219	619 ± 215	708 ± 255	598 ± 242	697 ± 239
Frequency Domain										
VLF_peak_ (Hz)	0.037 ± 0.003	0.035 ± 0.005	0.037 ± 0.005	0.037 ± 0.004	0.035 ± 0.004	0.035 ± 0.004	0.036 ± 0.003	0.035 ± 0.005	0.037 ± 0.003	0.036 ± 0.005
LF_peak_ (Hz)	0.1 ± 0.0	0.1 ± 0.0	0.1 ± 0.0	0.1 ± 0.0	0.1 ± 0.0	0.1 ± 0.0	0.1 ± 0.0	0.1 ± 0.0	0.1 ± 0.0	0.1 ± 0.0
HF_peak_ (Hz) ^a^	0.21 ± 0.03	0.20 ± 0.04	0.21 ± 0.03	0.20 ± 0.01	0.22 ± 0.04	0.19 ± 0.02	0.21 ± 0.04	0.19 ± 0.01	0.21 ± 0.02	0.20 ± 0.02
Non-linear										
SI ^b^	5.2 ± 2.4	4.6 ± 2.5	4.8 ± 1.9	3.9 ± 1.4	4.4 ± 2.2	3.9 ± 1.7	4.0 ± 1.6	3.5 ± 1.3	4.3 ± 2.0	3.5 ± 1.3
SD1 (%) ^a^	34.3 ± 4.9	35.0 ± 3.8	34.5 ± 5.6	33.9 ± 4.0	33.7 ± 3.1	34.8 ± 3.8	34.2 ± 4.1	35.0 ± 3.6	34.5 ± 4.6	36.2 ± 5.4
SD2 (%) ^a^	65.7 ± 4.9	65.2 ± 3.8	65.5 ± 5.6	66.1 ± 4.0	66.3 ± 3.1	65.2 ± 3.8	65.9 ± 4.1	65.0 ± 3.6	65.5 ± 4.6	63.9 ± 5.4
SD2/SD1 ^b^	1.96 ± 0.37	1.89 ± 0.30	1.96 ± 0.37	1.98 ± 0.33	1.99 ± 0.26	1.91 ± 0.29	1.97 ± 0.35	1.89 ± 0.29	1.94 ± 0.36	1.82 ± 0.41

Data are mean ± SD. Data were collected over 10 min during paced breathing. R-to-R_int_: R-to-R interval. SDNN: Standard deviation of N-to-N intervals. RMSSD: Root mean square of successive R-to-R interval differences. R-to-R Triangular Index: Integral of the density of the R-to-R interval histogram divided by its height. TINN: Baseline width of the R-to-R interval histogram. VLF_peak_: Peak frequency of the very low frequency band (0.0033–0.04 Hz). LF_peak_: Peak frequency of the low frequency band (0.04–0.15 Hz). HF_peak_: Peak frequency of the high frequency band (0.15–0.4 Hz). SI: Stress Index. SD1: Poincaré plot standard deviation perpendicular the line of identity. SD2: Poincaré plot standard deviation along the line of identity. SD2/SD1: Ratio of SD2-to-SD1. ^a^ Denotes main effect of time (*p* < 0.05). ^b^ Denotes non-significant effect of time (0.05 < *p* < 0.08). There were no time x CBD preparation interactions (all *p* > 0.10). Circulating CBD concentrations for preparations 178 and 203 were below the limit of quantitation at 60 min, thus heart rate variability data associated with these preparations were excluded from statistical analysis.

**Table 5:** Heart rate and blood pressure response to five different cannabidiol preparations.

Time	Variable	Cannabidiol Preparation (30 mg)				
		178	203	340	472	707
Base	HRBP	53 ± 8 115/70 ± 9/6	58 ± 9 116/67 ± 12/8	56 ± 16 115/71 ± 8/6	54 ± 10 115/70 ± 9/6	54 ± 10 115/72 ± 11/8
30	HRBP	57 ± 9 121/70 ± 11/8	58 ± 9 120/71 ± 12/5	54 ± 10 117/72 ± 11/7	56 ± 12 117/70 ± 12/9	55 ± 11 121/69 ± 12/9
60	HRBP	58 ± 9 119/72 ± 10/6	56 ± 10 119/68 ± 5/7	54 ± 9 118/71 ± 10/7	55 ± 10 119/69 ± 11/5	55 ± 12 120/71 ± 11/8
120	HRBP	64 ± 10 125/68 ± 9/5	65 ± 11 122/70 ± 11/6	62 ± 10 124/68	64 ± 10 118/66 ± 10/7	64 ± 12 125/69 ± 9/7
180	HRBP	65 ± 10 123/66 ± 12/9	66 ± 11 121/68 ± 10/8	65 ± 12 122/66 ± 12/7	64 ± 11 123/68 ± 11/9	63 ± 10 120/67 ± 12/7
240	HRBP	65 ± 12 124/66 ± 9/5	67 ± 10 125/67 ± 9/4	64 ± 13 122/67 ± 7/8	66 ± 11 124/66 ± 11/7	63 ± 9 119/68 ± 12/8

HR: Heart Rate (beats/minute). BP: Blood pressure (mmHg). Data are mean and SD.

**Table 6:** Comparison of pharmacokinetic parameters of current preparations with previously reported studies of ingestible cannabidiol.

Study	Formulation, Administration, CBD Single Dose (mg)	T_max_ (h)	C_max_ (ng/mL)	AUC_0-t_ (h × ng/mL)	AUC_0-inf_ (h × ng/mL)	t_½_ (h)	K_a_ (1/h)	K_e_ (1/h)	V_d_ (L)
[26]	Oral capsule (CBD + THC) 5.4 mg	0.99	0.93	4.35					
[27]	Oral capsule (CBD + THC) 5.4 mg	1.0	0.95						
[28]	GW oral capsule (CBD + THC) 10 mg	1.27	2.47	5.76	6.03	1.09			
[29]	Oral capsule (CBD + THC) 10 mg	1	2.1	6.9					
[21]	PTL101 * CBD oral capsule 10 mg	3	3.22	9.64	10.31	2.95		0.1	
[30]	PTL401 * (CBD + THC) oral capsule 10 mg	1.25	2.94	9.85	10.52	3.21		0.29	
[18]	Oral capsule MCT-CBD ** 25 mg	3.0	3.05	9.51	19.23				
[18]	Oral capsule SEEDS-CBD *** 25 mg		13.53	27.15	32.63				
[12]	Caliper CBD **** water soluble 30 mg	0.9	2.82	6.80	7.94	2.54	1.68	0.66	
[12]	Caliper CBD lipid soluble 30 mg	1.5	0.65	1.51	1.64	2.30	1.14	0.72	
	Preparation 178 CBD 30 mg	3.29	2.20	4.58	-	-	0.32	-	-
	Preparation 203 CBD 30 mg	3.39	1.29	2.30	-	-	0.24	-	-
	Preparation 340 CBD 30 mg	1.28	3.54	7.81	13.81	2.20	1.19	0.40	7428
	Preparation 472 CBD 30 mg	1.53	2.88	6.32	9.96	5.18	1.87	0.27	20,178
	Preparation 707 CBD 30 mg	0.70	5.57	9.12	10.77	1.42	1.43	0.56	8024
[21]	PTL101 CBD oral capsule 100 mg	3.5	47.44	150	153	3.59			
[31]	(Epidiolex^®^) 200 mg	2.3	148.0	449	474	8.58			4105
[32]	(Epidiolex^®^) 200 mg	2.5	200.0	671	600	15.5			6661
[32]	(Epidiolex^®^) 200 mg	2.0	172.0	530	522	14.6			7778
[32]	(Epidiolex^®^) 200 mg	2.5	155.0	532	601	13.1			6016
[32]	(Epidiolex^®^) 200 mg	2.5	153.0	464	499	11.2			5800
[33]	GW oral CBD capsule 400 mg	3	181.2	704					
[33]	GW oral CBD capsule 400 mg	1.5	114.2	482					
[34]	(Epidiolex^®^) 750 mg	4.0	187.0	1077	1190	39.7			
[33]	GW oral CBD capsule 800 mg	3	221.1	867					
[33]	GW oral CBD capsule 800 mg	4	157.1	722					
[35]	GW oral solution 1500 mg	4	292.4	1517	1618	14.43			20,963
[35]	GW oral solution 1500 mg	3.5	335.4	1987	2198	30.33			34,101
[36]	(Epidiolex^®^) 1500 mg	6.13	524.5	2650	2713				
[35]	GW oral solution 3000 mg	5	533.0	2669	2802	14.39			23,357
[35]	GW oral solution 4500 mg	5	722.1	3215	3426	16.61			36,575
[36]	(Epidiolex^®^) 4500 mg	4.07	426.9	2339					
[35]	GW oral solution 6000 mg	5	782	3696	3900	15.42			42,849

CBD: Cannabidiol. T_max_: the time to maximum concentration. C_max_: the maximum concentration. AUC_0-t_: the area under the curve representing total cannabidiol exposure between 0 and end of data collection. AUC_inf_: an estimate of the total exposure to cannabidiol over time. t_½_: the amount of time it takes to decrease the circulating concentration to half of its initial value. K_a_: the rate at which the cannabidiol is absorbed into the body. K_e_: the rate at which the cannabidiol is removed from the body. V_d_: the volume of distribution, an estimate of the degree to which cannabidiol is distributed in the body tissue vs. the plasma. The information presented in this table represents an update to a previous review [19]. * PTL- gelatin matrix pellets technology-based formulation; ** MCT-CBD—medium-chain triglycerides; *** SEEDS-CBD—self-emulsifying drug delivery system. **** Caliper CBD Water Soluble is identical to Preparation 472. The background color highlights the preparations investigated in the current study.

## Data Availability

The data presented in this study are available as supplementary material.
